# Copper and cuproptosis-related genes in hepatocellular carcinoma: therapeutic biomarkers targeting tumor immune microenvironment and immune checkpoints

**DOI:** 10.3389/fimmu.2023.1123231

**Published:** 2023-04-20

**Authors:** Xiaoqiang Wang, Dongfang Chen, Yumiao Shi, Jiamei Luo, Yiqi Zhang, Xiaohong Yuan, Chaojin Zhang, Huigang Shu, Weifeng Yu, Jie Tian

**Affiliations:** ^1^ Department of Anesthesiology, Renji Hospital, Shanghai Jiaotong University School of Medicine, Shanghai, China; ^2^ Department of Anesthesiology, Shanghai Fifth People’s Hospital, Fudan University, Shanghai, China; ^3^ Zhejiang Cancer Hospital, Institute of Basic Medicine and Cancer (IBMC), Chinese Academy of Sciences, Hangzhou, China

**Keywords:** copper, cuproptosis, hepatocellular carcinoma, immune checkpoints, tumor immune microenvironment

## Abstract

**Background:**

Hepatocellular carcinoma (HCC), one of the most common cancers worldwide, exhibits high immune heterogeneity and mortality. Emerging studies suggest that copper (Cu) plays a key role in cell survival. However, the relationship between Cu and tumor development remains unclear.

**Methods:**

We investigated the effects of Cu and cuproptosis-related genes (CRGs) in patients with HCC in the TCGA-LIHC (The Cancer Genome Atlas-Liver cancer, *n* = 347) and ICGC-LIRI-JP (International Cancer Genome Consortium-Liver Cancer-Riken-Japan, *n* = 203) datasets. Prognostic genes were identified by survival analysis, and a least absolute shrinkage and selection operator (Lasso) regression model was constructed using the prognostic genes in the two datasets. Additionally, we analyzed differentially expressed genes and signal pathway enrichment. We also evaluated the effects of CRGs on tumor immune cell infiltration and their co-expression with immune checkpoint genes (ICGs) and performed validation in different tumor immune microenvironments (TIMs). Finally, we performed validation using clinical samples and predicted the prognosis of patients with HCC using a nomogram.

**Results:**

A total of 59 CRGs were included for analysis, and 15 genes that significantly influenced the survival of patients in the two datasets were identified. Patients were grouped by risk scores, and pathway enrichment analysis suggested that immune-related pathways were substantially enriched in both datasets. Tumor immune cell infiltration analysis and clinical validation revealed that PRNP (Prion protein), SNCA (Synuclein alpha), and COX17 (Cytochrome c oxidase copper chaperone COX17) may be closely correlated with immune cell infiltration and ICG expression. A nomogram was constructed to predict the prognosis of patients with HCC using patients’ characteristics and risk scores.

**Conclusion:**

CRGs may regulate the development of HCC by targeting the TIM and ICGs. CRGs such as PRNP, SNCA, and COX17 could be promising targets for HCC immune therapy in the future.

## 1 Background

Cancer is one of the leading causes of death worldwide and places a heavy burden on global health (1). According to statistical reports, hepatocellular carcinoma (HCC) is currently the third most common cancer worldwide. Moreover, based on related reports, >45% of new HCC cases and related deaths occurred in China (2–4). Although there have been advances in HCC therapy in recent years, the high heterogeneity and lack of accurate early diagnostic biomarkers have resulted in the poor prognosis of patients with HCC (5).

For patients with advanced HCC, immunotherapy has emerged as a prospective therapeutic approach through the targeting of programmed cell death protein 1 (PD-1)/programmed cell death ligand 1 (PD-L1) and cytotoxic T lymphocyte antigen 4 (CTLA4) (6). Studies have suggested that the objective response rates of anti-PD-1 treatment (including nivolumab, pembrolizumab, and camrelizumab) increased to about 15%–20% for patients with HCC that were pretreated with sorafenib (7–9). However, drugs targeting PD-1 and PD-L1 benefit few patients with HCC, as most patients have poor responses to immune checkpoint inhibitors (ICIs) (10). This may be attributed to the intrinsically high heterogeneity and immune suppression microenvironment of HCC (10, 11). Previous studies demonstrated that a large number of suppressive immune cells, such as tumor-associated macrophages (TAMs), myeloid-derived suppressor cells (MDSCs), and regulatory T cells (Tregs), were recruited to the tumor microenvironment of HCC, resulting in immune cell dysfunction and immune surveillance escape (12, 13). Therefore, exploring effective targets to improve HCC patients’ response to ICIs is important.

Copper (Cu) is an endogenous metal essential for all living organisms and participates in various biological functions, such as mitochondrial respiration, iron uptake, redox reactions, glucose regulation, and cholesterol metabolism (14, 15). However, excessive accumulation of Cu induces oxidative stress, cytotoxicity, or even cuproptosis. The latter is a type of cell death that is regulated by Cu and mitochondrial respiration, which has been recently discovered (16). Furthermore, the dysfunction of Cu homeostasis can lead to severe disorders such as Wilson’s and Menke’s diseases (17). Therefore, intracellular Cu concentrations are typically strictly maintained at extraordinarily low levels *via* complex homeostatic mechanisms. Exploring the mechanisms underlying Cu homeostasis dysfunction and imbalanced cuproptosis may aid in the identification of novel therapeutic targets for various diseases.

A previous study has shown a significant increase in Cu levels in patients with cancer compared with healthy individuals (18). For instance, a meta-analysis including 36 studies revealed significantly upregulated serum Cu levels in patients with breast cancer compared with healthy controls (19). Furthermore, some studies have demonstrated the effective antitumor effects of Cu ionophores such as elesclomol (16, 20, 21). Some studies have also found associations among cuproptosis, tumor development, and response to ICIs (22, 23). For instance, Luo et al. (24) found that cuproptosis could regulate the response of acute myeloid leukemia cells to the immune system. Xiong et al. (25) suggested that cuproptosis may be regulated by p53, a crucial tumor suppressor and metabolic regulator. Thus, targeting cuproptosis may be a promising strategy for HCC immunotherapy.

Studies on the role of Cu and cuproptosis-related genes (CRGs) in HCC are lacking. Herein, we systematically analyzed the functions and effects of CRGs on the survival of patients with HCC based on two public HCC datasets. We aimed to identify the critical CRGs that significantly influence the overall survival (OS) of patients with HCC and to construct a useful nomogram to predict the prognosis of patients. Moreover, we investigated the relationships among CRGs, tumor immune cell infiltration, and immune checkpoint genes (ICGs) to detect potential HCC biomarkers targeting the tumor immune microenvironment (TIM).

## 2 Methods

### 2.1 Data acquisition and CRG list

The total transcriptome RNA sequencing (RNA-seq) data and clinical information of patients with HCC were obtained and downloaded from The Cancer Genome Atlas Liver Hepatocellular Carcinoma (TCGA-LIHC) dataset (https://tcga-data.nci.nih.gov/tcga/), the International Cancer Genome Consortium (ICGC) portal (https://dcc.icgc.org/projects/LIRI-JP), and GEO datasets (https://www.ncbi.nlm.nih.gov/). The list of CRGs and their and functions were obtained from the Gene Ontology (GO) resource (http://geneontology.org/) and a published paper (16). The full list of CRGs is provided in **Supplementary Table 1**.

### 2.2 Survival analysis

The effects of CRGs on the OS of patients with HCC were validated using survival analysis. Patients were categorized into the low-expression (L) and high-expression (H) groups, and the median gene expression level was chosen as the cutoff value. Similarly, survival analysis of the risk scores obtained from the least absolute shrinkage and selection operator (Lasso) regression model was performed, and patients were assigned to the low-risk or high-risk group based on their risk scores. The cutoff value for grouping was the median risk score. The survival analysis was performed using the “survminer” R package.

### 2.3 Construction of the Lasso regression model

Prognostic genes with a *P-*value of <0.05 in the survival analysis in the two datasets were used to construct the model. A Lasso regression model (26) for predicting the prognosis of patients with HCC was constructed using the prognostic genes in the two datasets using the “glmnet” R package. A 10-fold cross-validation method was used to optimize the model. The risk score predicting the OS was calculated for every patient using the following formula: risk score = (gene A expression × a) + (gene B expression × b) … + (gene N expression × n), where a, b, and n represent regression coefficients.

### 2.4 Validation and effectiveness of the prognostic model

To validate the model’s effectiveness, survival and time-dependent receiver operating characteristic (ROC) curve analyses were performed based on the survival time, survival status, and risk scores of patients with HCC using the “survminer” and “pROC” R packages. Relationships among the risk scores, OS, survival status, and gene expression of selected CRGs were analyzed using the online bioinformatic analysis tool Sangerbox 3.0 (http://vip.sangerbox.com/home.html).

### 2.5 Differentially expressed gene analysis

Patients were grouped according to risk scores, and DEGs were identified using the “limma” R package. Briefly, genes with a false discovery rate (FDR) of <0.05 and fold change of >1.5 between the two groups were identified as DEGs. DEGs were visualized with a volcano plot and generated using Sangerbox 3.0 (http://vip.sangerbox.com/home.html).

### 2.6 Functional enrichment analysis

DEGs were used for multiple functional enrichment analyses including Gene Set Enrichment Analysis (GSEA) and Kyoto Encyclopedia of Genes and Genome (KEGG) pathway and Gene Ontology-Biological Process (GO-BP) enrichment analyses using the “clusterProfiler (version 3.14.3)” R package (27) and GSEA software (version 3.0, http://software.broadinstitute.org/gsea/index.jsp). The minimum and maximum number of genes in the cluster were 5 and 5000, respectively. Pathways with a *P*-value of <0.05 and FDR of <0.05 were considered statistically different.

Immune-related pathway enrichment (GO-immune system process) was analyzed using the Cytoscape software and ClueGO application (https://cytoscape.org/).

### 2.7 Tumor immune cell infiltration analysis

Tumor immune cell infiltration levels were evaluated using the TIMER method (28) and the “IOBR” R package in the TCGA database (29). Relationships between gene expression levels and immune cell infiltration levels were calculated using the “psych (version 2.1.6)” R package. Moreover, ESTIMATE analysis (https://bioinformatics.mdanderson.org/estimate/, including ESTIMATE score, stromal score, and immune score) was performed to visualize the correlations between screened CRGs and TME in the TCGA database. These analyses were performed using the open-source online tool Sangerbox 3.0 (http://vip.sangerbox.com/home.html).

### 2.8 Expression of ICGs and correlations with CRGs in HCC

The expression levels of *PDCD1* (the gene coding PD-1), *CD274* (the gene coding PD-L1), and *CTLA4* in normal and HCC liver tissues were analyzed using data obtained from UALCAN (http://ualcan.path.uab.edu/analysis.html) (30). Additionally, co-expression analysis between ICGs and CRGs in HCC was performed using data obtained from cBioportal (https://www.cbioportal.org/) and the Firehose Legacy dataset (31).

### 2.9 Myeloid response score and different immune subtypes in HCC

The myeloid response score (MRS) model was used as a reference to distinguish between the immune subtypes in HCC (32). RNA-seq data of patients with HCC with different MRSs was obtained and analyzed using data obtained from the GSE134921 dataset. Expression levels of critical CRGs were compared between the high-MRS and low-MRS groups.

### 2.10 Construction of a prognostic nomogram for HCC

To provide a reliable and quantifiable method to predict the prognosis of patients with HCC, a novel nomogram was constructed by integrating risk score, age, sex, race, TNM (tumor, nodes, metastases) stage, and tumor grade into a Cox regression model using the “rms” R package.

### 2.11 Recruitment of patients with HCC and collection of clinical HCC samples

An observational study was conducted at the Renji Hospital, Shanghai Jiaotong University School of Medicine, and Eastern Hepatobiliary Surgery Hospital, the Third Affiliated Hospital of Naval Medical University. This study was approved by the Renji Hospital Ethics Committee (KY2020-185). The study complied with the Declaration of Helsinki and the Consolidated Standards of Reporting Trials (CONSORT) statement. Patients aged ≥18 years, those with primary HCC, and those who received HCC excision surgery were included in the study. Patients were excluded if they suffered from multiple metastases, had other additional types of cancer, or had missing clinical data. HCC samples were collected in the operation room immediately after excision and stored at −80°C. All samples were confirmed as HCC by pathological diagnosis after surgery.

### 2.12 Expression levels of ICGs and CRGs in HCC samples

Gene expression levels of ICGs (*PDCD1*, *CD274*, and *CTLA4*) and CRGs (*PRNP*, *SNCA*, *COX17*, *ATP7A*, *ATP13A2*, and *F5*) were analyzed in human HCC samples. Total RNA was extracted from the HCC samples using the EZ-press RNA Purification Kit (EZ Bioscience, USA) according to the manufacturers’ protocol. The primers of genes are listed in **Supplementary Table 2**. Linear correlations between the gene expression levels of ICGs and CRGs were analyzed.

### 2.13 Immunohistochemical staining of ICGs and CRGs in HCC samples

To determine the protein expression levels of ICGs and CRGs in HCC samples, IHC staining of PD-1 (Servicebio, cat: GB11338-1), PD-L1 (Servicebio, cat: GB11339A), PRNP (Abclonal, cat: A18058), SNCA (Abclonal, cat: A20407), and COX17 (SANTA Cruz, cat: sc-100521) was performed.

### 2.14 Statistical analyses

Statistical analyses were performed using IBM SPSS Statistics 23.0 (SPSS Inc., Armonk, NY, USA). Differences in the survival analysis were compared by log-rank t-test with a 95% confidence interval. ROC curves were plotted and area under the curve (AUC) values were calculated to assess the discrimination strength of the model. Linear correlations were assessed using Spearman’s or Pearson’s correlation tests, and the correlation coefficient “r” was calculated. All statistical tests were two sided. A *P*-value of <0.05 was considered statistically significant.

## 3 Results

The study design flow chart and validation process is presented in **Supplementary Figure 1**. A total of 59 out of 62 CRGs were analyzed in the two datasets because the expression data of three CRGs (*MT1HL1*, *MT-CO1*, and *MT-CO2*) was missing from the raw data. From the TCGA-LIHC and ICGC-LIRI-JP datasets, 347 and 203 patients with HCC were examined, respectively.

### 3.1 Screening of prognostic genes in the TCGA-LIHC and ICGC-LIRI-JP datasets

**Figure 1:**
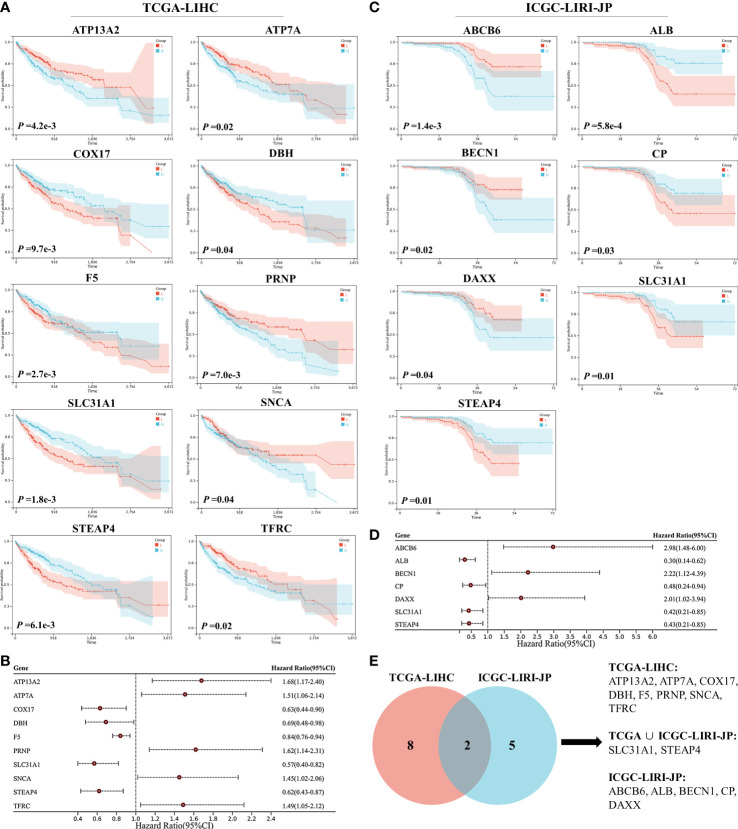
Survival analyses of cuproptosis-related genes (CRGs) for patients with hepatocellular carcinoma (HCC) in the TCGA-LIHC and ICGC-LIRI-JP datasets. **(A)** Survival analyses of CRGs for patients with HCC in the TCGA-LIHC dataset. **(B)** Forest plot of prognostic genes in the TCGA-LIHC dataset. **(C)** Survival analyses of CRGs for patients with HCC in the ICGC-LIRI-JP dataset. **(D)** Forest plot of prognostic genes in the ICGC-LIRI-JP dataset. **(E)** Venn diagram of prognostic genes in the two datasets.

Survival analysis was performed on 59 CRGs in the TCGA-LIHC dataset and 10 prognostic genes (*ATP13A2*, *ATP7A*, *COX17*, *DBH*, *F5*, *PRNP*, *SLC31A1*, *SNCA*, *STEAP4*, and *TFRC*) that were significantly correlated with the OS of patients were identified (**Figure 1A**). Among these 10 critical genes, *ATP13A2*, *ATP7A*, *PRNP*, *SNCA*, and *TFRC* were unfavorable for patient OS, whereas *COX17*, *DBH*, *F5*, *SLC31A1*, and *STEAP4* were favorable for patients’ prognosis (**Figure 1B**). Similarly, seven genes (*ABCB6*, *ALB*, *BECN1*, *CP*, *DAXX*, *SLC31A1*, and *STEAP4*) were found to significantly influence the OS of patients in the ICGC-LIRI-JP dataset, and higher expression levels of *ABCB6*, *BECN1*, and *DAXX* were associated with worse OS, whereas those of *ALB*, *CP*, *SLC31A1*, and *STEAP4* were associated with better prognosis for patients with HCC (**Figures 1C, D**). Altogether, 15 prognostic genes were identified in the two datasets (**Figure 1E**).

### 3.2 Lasso model construction and validation

**Figure 2:**
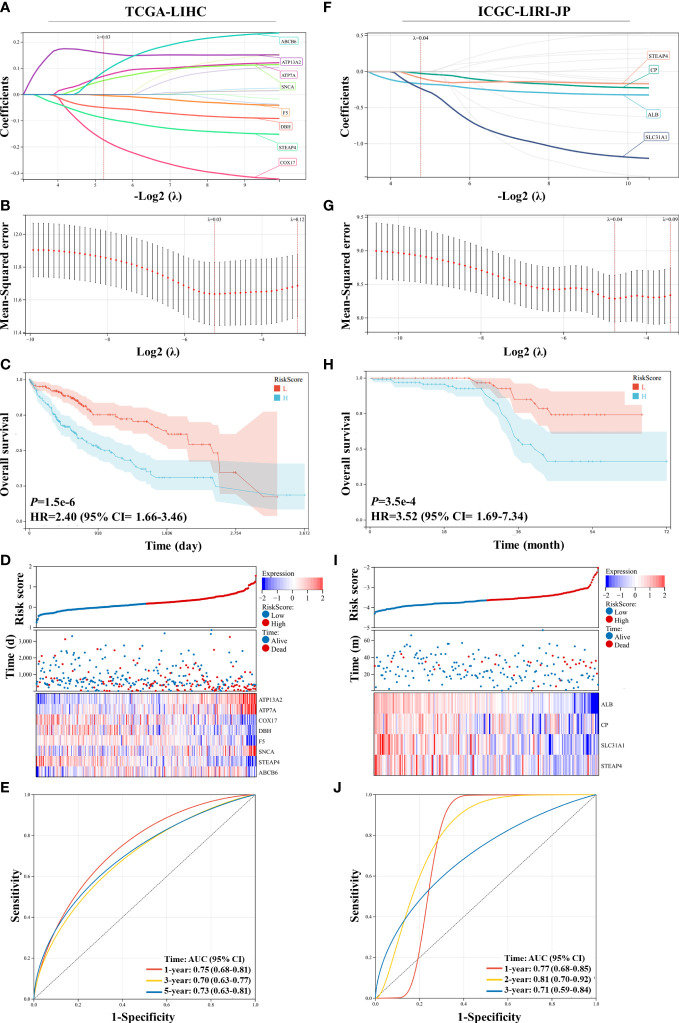
Construction and validation of the least absolute shrinkage and selection operator (Lasso) model in the TCGA-LIHC and ICGC-LIRI-JP datasets. **(A, B)** The Lasso regression model was constructed using 15 prognostic genes in the TCGA-LIHC dataset, and eight genes were successfully included in the model. **(C)** Kaplan–Meier survival analysis of patients with hepatocellular carcinoma (HCC) grouped by risk scores in the TCGA-LIHC dataset. **(D)** Distribution of the risk scores, survival status, and expression of eight critical predictive genes. **(E)** Receiver operating characteristic (ROC) curve of risk scores in the TCGA-LIHC dataset. **(F, G)** The Lasso regression model was constructed using 15 prognostic genes in the ICGC-LIRI-JP dataset, and four genes were successfully included in the model. **(H)** Kaplan–Meier survival analysis of patients with HCC grouped by risk scores in the ICGC-LIRI-JP dataset. **(I)** Distribution of the risk scores, survival status, and expression of four critical predictive genes. **(J)** ROC curve of risk scores in the ICGC-LIRI-JP dataset.

A Lasso regression model was constructed using the 15 prognostic genes identified above. Eight genes were successfully included in the model from the TCGA-LIHC dataset; the formula used was follows: risk score = 0.158 × ATP13A2 + 0.070 × ATP7A − 0.173 × COX17 − 0.050 × DBH − 0. 004 × F5 + 0.054 × SNCA − 0.089 × STEAP4 + 0.087 × ABCB6 (**Figures 2A, B**). Patients were assigned to the low-risk or high-risk group based on the median of all the risk scores. Survival analysis revealed that patients in the high-risk group showed reduced survival years compared with patients in the low-risk group, with the hazard ratio reaching 2.40 (**Figure 2C**). The heatmap also demonstrated that more deaths were observed in the high-risk group (**Figure 2D**). ROC curve analysis revealed moderate predictive efficacy, with the AUC for 1-year survival prediction reaching 0.75 (**Figure 2E**). Similar model construction was conducted for the ICGC-LIRI-JP dataset, and four critical genes (*ALB*, *CP*, *SLC31A1*, and *STEAP4*) were included in the model (**Figures 2F, G**). Survival analysis and heatmaps revealed significantly increased survival years and fewer patient deaths in the low-risk group compared with the high-risk group (**Figures 2H, I**). The AUC for 1-year and 2-year survival prediction reached 0.77 and 0.81 respectively, suggesting good predictive effects of the model (**Figure 2J**).

### 3.3 DEG validation and potential immune-related pathway enrichment

**Figure 3:**
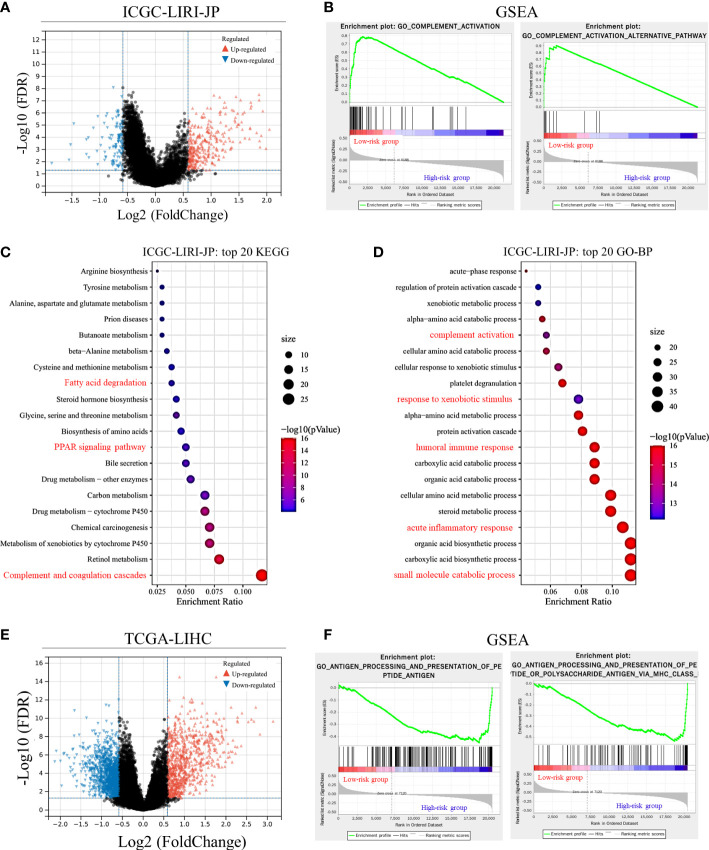
Differentially expressed gene (DEG) validation and potential pathway enrichment analysis. **(A)** Volcano plot of DEGs in the ICGC-LIRI-JP dataset. **(B)** Gene Set Enrichment Analysis (GSEA) of immune-related pathways in the ICGC-LIRI-JP dataset. **(C, D)** Top 20 enriched Kyoto Encyclopedia of Genes and Genomes (KEGG) pathways and Gene Ontology-biological process (GO-BP) pathways in the ICGC-LIRI-JP dataset. **(E)** Volcano plot of DEGs in the TCGA-LIHC dataset. **(F)** GSEA of immune-related pathways in the TCGA-LIHC dataset.

DEGs between the two groups divided by the median risk score in the ICGC-LIRI-JP dataset were identified and are shown in **Figure 3A**. A total of 317 upregulated and 113 downregulated DEGs were identified. GSEA showed that immune-related pathways, including complement activation and complement activation alternative pathway, were significantly different between the two groups. Additionally, complement activation-related genes were downregulated in the high-risk group compared with the low-risk group (**Supplementary Figure 2**). KEGG and GO-BP pathway enrichment analyses revealed considerable changes in immune-related pathways, including complement-related signal pathways, humoral immune response, and response to xenobiotic stimulus (**Figures 3B–D**). In the TCGA-LIHC dataset, 812 upregulated DEGs and 1333 downregulated DEGs were identified (**Figure 3E**). GSEA revealed that immune-related pathways, such as antigen processing and presentation pathways, were enriched in the TCGA-LIHC dataset (**Figure 3F**). KEGG pathway enrichment analysis showed that the complement and coagulation cascade pathways were significantly enriched (**Supplementary Figure 3**), suggesting potential associations between Cu homeostasis and immune function. Interestingly, metabolic pathways, such as small molecule catabolic processes and fatty acid metabolism, were also significantly enriched in both datasets, suggesting that CRGs play a role in cell metabolism (**Figures 3C, D** and **Supplementary Figure 3**) (33).

**Figure 4:**
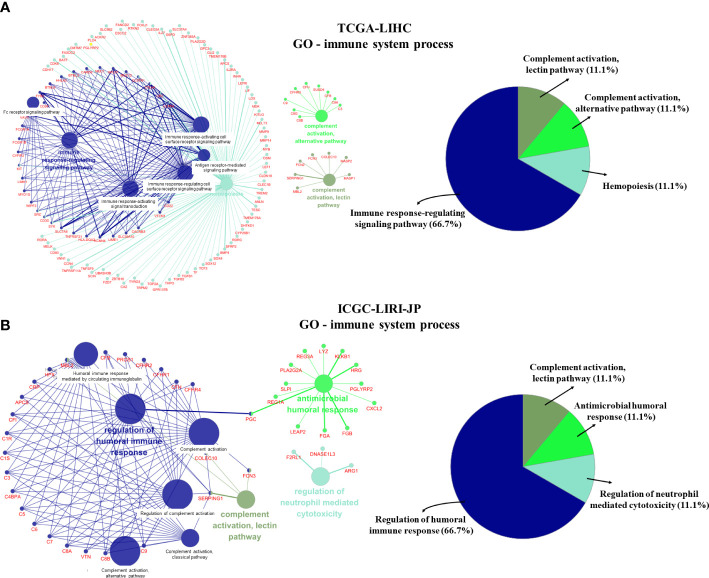
GO-immune system process pathway enrichment using differentially expressed genes (DEGs). **(A)** GO-immune system process pathway enrichment in the TCGA-LIHC dataset. **(B)** GO-immune system process pathway enrichment in the ICGC-LIRI-JP dataset.

We further visualized the enrichment of GO-immune system process pathways using DEGs from the two datasets (**Figure 4**). Following enrichment, immune-related signal pathways were found to be considerably altered between the two groups. In the TCGA-LIHC dataset, DEGs were enriched mainly in the immune response-regulatory signaling pathway (66.7%), complement activation (11.1%), complement activation alternative pathway (11.1%), and hemopoiesis (11.1%) (**Figure 4A**). In the ICGC-LIRI-JP dataset, regulation of humoral immune response (66.7%), complement activation-lectin pathway (11.1%), antimicrobial humoral response (11.1%), and regulation of neutrophil-mediated cytotoxicity (11.1%) were significantly enriched (**Figure 4B**). These findings indicate the potential role of CRGs in immune function/response regulation in HCC.

### 3.4 Correlations between CRGs and tumor immune cell infiltration

**Figure 5:**
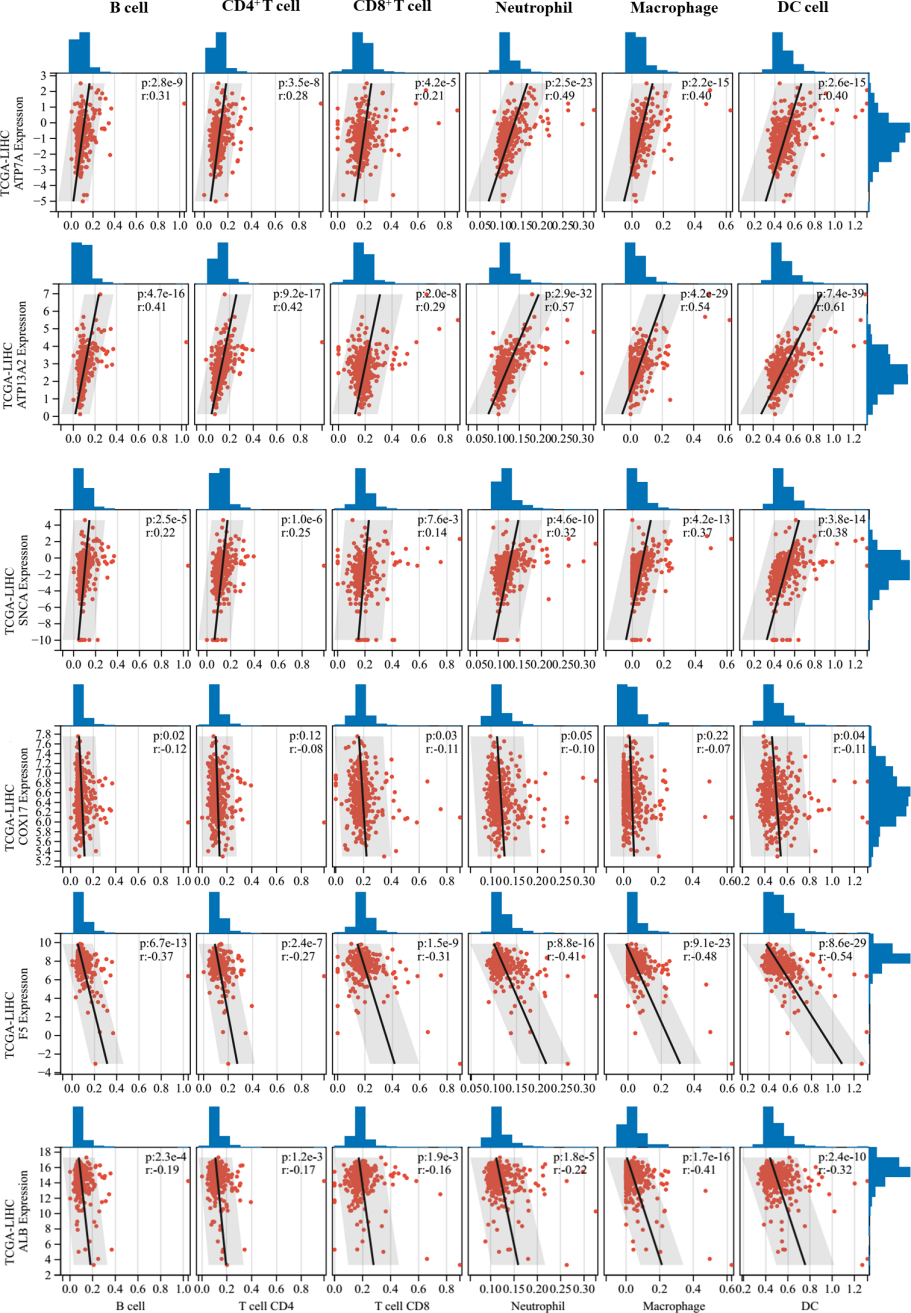
Correlations between critical cuproptosis-related genes (CRGs) and tumor immune cell infiltration. ATP7A, ATP13A2, and SNCA were positively correlated with multiple types of immune cell infiltration in hepatocellular carcinoma (HCC), while COX17, F5, and ALB were negatively correlated with immune cell infiltration in HCC.

Correlations between 11 prognostic genes screened by Lasso models in the two datasets and tumor immune cell infiltration levels were analyzed using the TIMER method. Six CRGs that were significantly correlated with tumor immune cell infiltration levels were identified (**Figure 5**). Interestingly, *ATP7A*, *ATP13A2*, and *SNCA*, which were all unfavorable for the OS of patients (**Figure 1**), were found to be positively correlated with multiple types of immune cell infiltration in HCC, whereas *COX17*, *F5*, and *ALB*, which were all favorable for the OS of patients, were negatively correlated with immune cell infiltration in HCC. Furthermore, we performed ESTIMATE analysis (including ESTIMATE score, stromal score, and immune score) to picture the correlations between the screened CRGs and tumor microenvironment (**Supplementary Table 3**). *ATP7A*, *ATP13A2*, *PRNP*, and *SNCA* were found to be positively correlated with the three scores, whereas *COX17* and *F5* were found to be negatively correlated with the three scores. No significant correlations were found between *ALB* and the three scores.

### 3.5 Correlations between CRGs and ICGs

**Figure 6:**
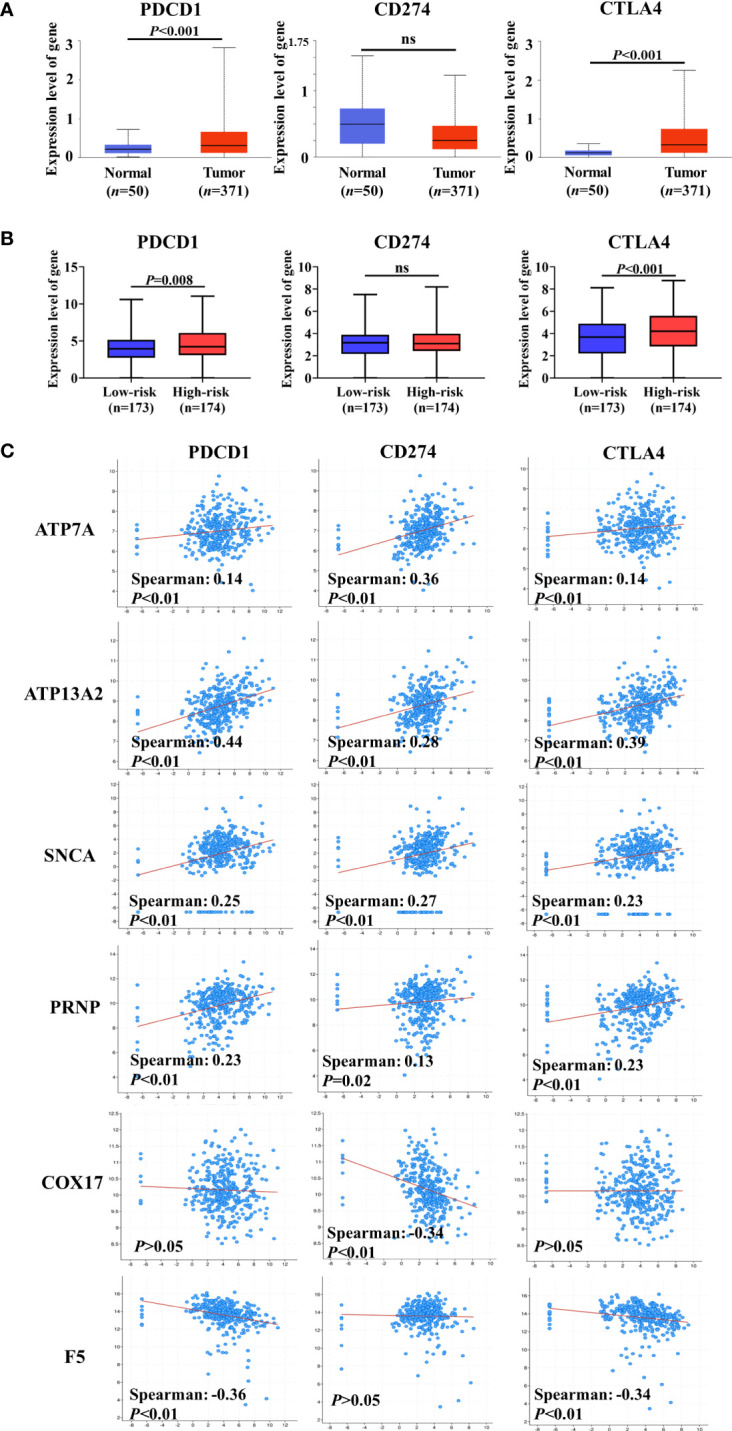
Co-expression analysis of cuproptosis-related genes (CRGs) and immune checkpoint genes (ICGs). **(A)** Expression levels of ICGs between normal liver tissue and hepatocellular carcinoma (HCC). **(B)** Differences in the expression of ICGs between low-risk and high-risk groups. **(C)** Co-expression analysis of CRGs and ICGs. ATP7A, ATP13A2, and SNCA were significantly positively correlated with the expression of ICGs, and a negative correlation between ICGs and COX17 or F5 was observed. ns, no significance.

A previous study has indicated that intratumoral Cu and CRGs modulate the expression of ICGs (34). ICGs are widely expressed in diverse cancer cells, including HCC, and regulate tumor development (35, 36). Therefore, we compared expression levels of ICGs between normal and HCC liver tissues. Three ICGs were expressed in HCC, and the gene expression levels of *PDCD1* and *CTLA4* were substantially increased in HCC samples compared with normal liver tissue (**Figure 6A**). By grouping patients based on the median risk score, similar changes were observed in that the expression levels of *PDCD1* and *CTLA4* were significantly higher in the high-risk group than in the low-risk group (**Figure 6B**). These findings indicate that higher expression levels of ICGs may be correlated with worse prognosis in patients with HCC.

We assessed the co-expression between CRGs and ICGs at the mRNA level in HCC (**Figure 6C**). Notably, *ATP7A*, *ATP13A2*, *SNCA*, and *PRNP* (unfavorable for the OS of patients with HCC) were significantly positively correlated with the expression of ICGs. However, a negative correlation was observed between ICGs and *COX17* or *F5* (favorable for the OS of patients with HCC). Collectively, these results further suggest that CRGs participate in the regulation of ICG expression and tumor immune escape.

### 3.6 Validation of critical CRGs in different immune subtypes of HCC

**Figure 7:**
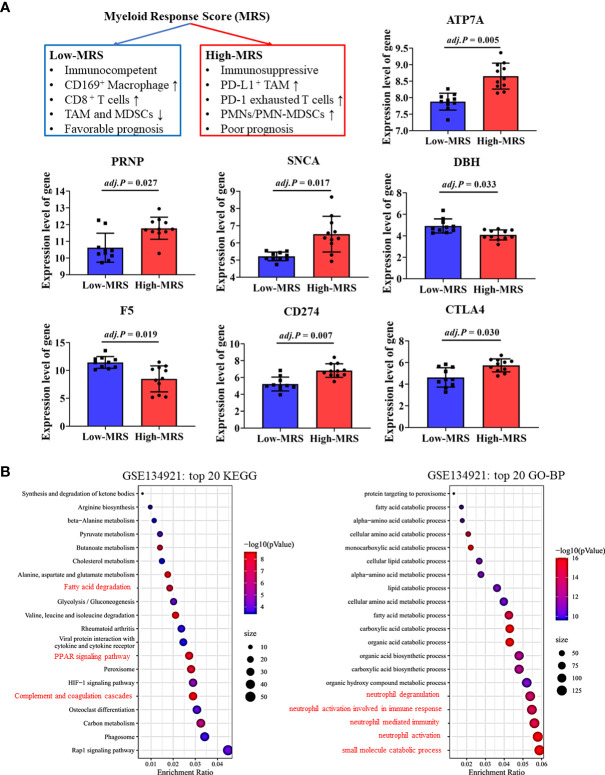
Validation of cuproptosis-related genes (CRGs) and immune checkpoint gene (ICG) expression in different immune subtypes of hepatocellular carcinoma (HCC). **(A)** Expression levels of critical CRGs and ICGs between the low-myeloid response score (MRS) group and high-MRS group. **(B)** Differential signal pathway analysis between the low- and high-MRS groups.

Recently, Wu et al. developed and validated a simple scoring model named MRS to distinguish between the different immune subtypes of HCC (32). A higher MRS usually represents a significantly immunosuppressive tumor microenvironment in HCC (**Figure 7A**). We validated our findings regarding the relationship between CRGs and the TIM using related sequencing data (GSE134921) (32). The expression of CRGs including *ATP7A*, *PRNP*, and *SNCA*, which were unfavorable for the prognosis of patients with HCC, was significantly increased in the high-MRS group (**Figure 7A**). In contrast, the expression of *DBH* and *F5*, which were favorable for the prognosis of patients with HCC, was reduced in the high-MRS group. It was found that *CD274* and *CTLA4* were remarkably upregulated in the high-MRS group. All the abovementioned results suggest close relationships among CRGs, TIM, and immune checkpoints in HCC.

Differential signaling pathways were analyzed and compared between the low-MRS and high-MRS groups (**Figure 7B**). Interestingly, the results of this analysis were surprisingly similar to those of the pathway enrichment analyses of the low-risk and high-risk groups (**Figure 4C** and **Supplementary Figure 3**), suggesting good comparability between the MRS and risk scoring systems. The risk score based on CRGs may discriminate immune subtypes in HCC. Similarly, metabolic pathways such as small molecule catabolic process and fatty acid metabolism were also enriched, suggesting that cell metabolism is associated with TIM regulation in HCC (**Figure 7B**).

### 3.7 Validation of significant correlations among CRGs, TIM, and ICGs in the Renji cohort

**Figure 8:**
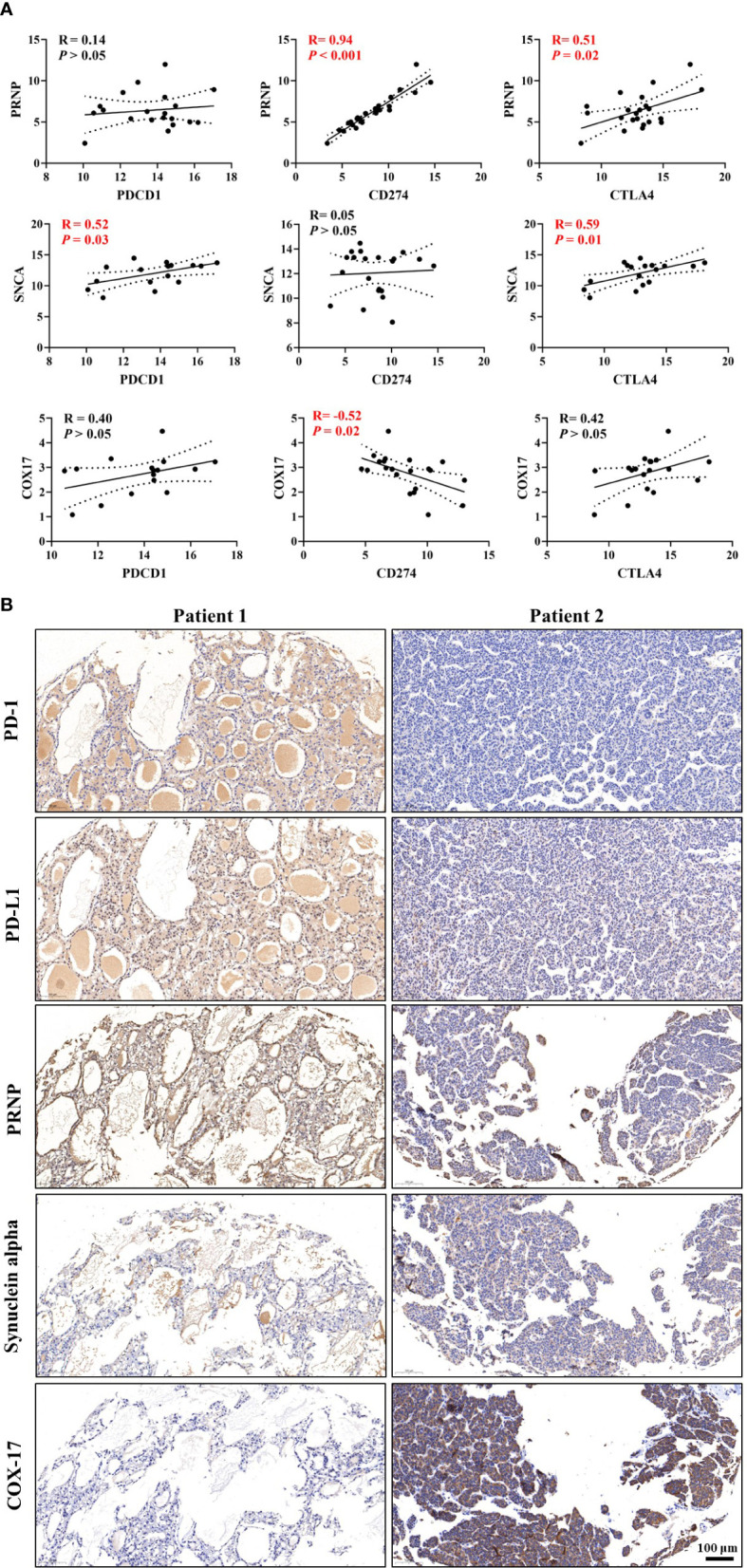
Validation of correlations among cuproptosis-related genes (CRGs), tumor immune microenvironment (TIM), and immune checkpoint genes (ICGs) in the Renji cohort. **(A)** Correlation analyses between CRGs and ICGs. **(B)** Immunohistochemical (IHC) staining of CRGs and ICGs in hepatocellular carcinoma (HCC) samples.

Based on correlations analyses between CRGs and ICGs (**Figure 8** and **Supplementary Figure 4**), we selected three CRGs (*PRNP* and *SNCA* [unfavorable for prognosis] and *COX17* [favorable for prognosis]) that had more significant correlations with ICGs than other CRGs for further validation in HCC samples; the clinical characteristics of patients are shown in **Table 1**. Linear regression analyses of gene expression levels revealed a significantly positive correlation between *PRNP* and ICGs, with the R-value reaching 0.94 and 0.51 for CD274 and CTLA4, respectively (**Figure 8A**). This strongly suggests that *PRNP* plays a key role in the regulation of ICGs in HCC. Positive correlations were observed between *SNCA* and *PDCD1*/*CTLA4*. However, a negative correlation was observed between *COX17* and *CD274*, with the R-value reaching −0.52 (**Figure 8A**). Moreover, IHC analysis of HCC samples revealed similar results (**Figure 8B**).

**Table 1:** Clinical characteristics of patients with HCC.

Characteristics	*n* = 25
Gender (male/female)	24/1
Age (year)	53.28 (11.05)
Height (cm)	170.43 (5.96)
Weight (kg)	68.10 (9.44)
ASA stage (I/II)	7/18
Child-Pugh stage (A/B)	13/12
Hypertension (Yes/No)	10/15
Diabetes (Yes/No)	2/23
Drinking (Yes/No)	5/20
Viral hepatitis (Yes/No)	24/1
Cirrhosis (Yes/No)	20/5
Tumor size (cm)	4.92 (2.53)
Operation time (hour)	2.80 (0.90)
Bleeding (ml)	295.45 (164.69)
Urine (ml)	413.64 (190.98)
Liquid transfusion	
Crystalloid fluid (ml)	1285.71 (373.21)
Colloid fluid (ml)	739.05 (375.53)
ALT (U/L)	36.64 (34.48)
AST (U/L)	35.05 (26.63)
Hb (g/L)	143.39 (16.43)
PLT (10^9^/L)	175.04 (86.76)
TBiL (μmol/L)	14.35 (5.76)
ALB (g/L)	42.98 (3.96)
Cr (μmol/L)	70.57 (13.15)
INR	1.02 (0.12)

Variables are shown as “mean (SD)”. ASA, American Society of Anesthesiologists; ALT, alanine transaminase; AST, aspartate aminotransferase; Hb, hemoglobin; PLT, platelets; TBiL, total bilirubin; ALB, albumin; Cr, creatine; INR, international normalized ratio.

Correlations between CRGs and immune cell markers were observed in HCC samples (**Supplementary Figure 5**). Linear regression analyses of gene expression levels suggested that *PRNP* be closely associated with multiple types of immune cells in HCC (**Supplementary Figure 5A**). In summary, the validations in HCC samples further verified the findings obtained from the comprehensive bioinformatic analyses.

### 3.8 Nomogram construction for HCC based on CRGs

**Figure 9:**
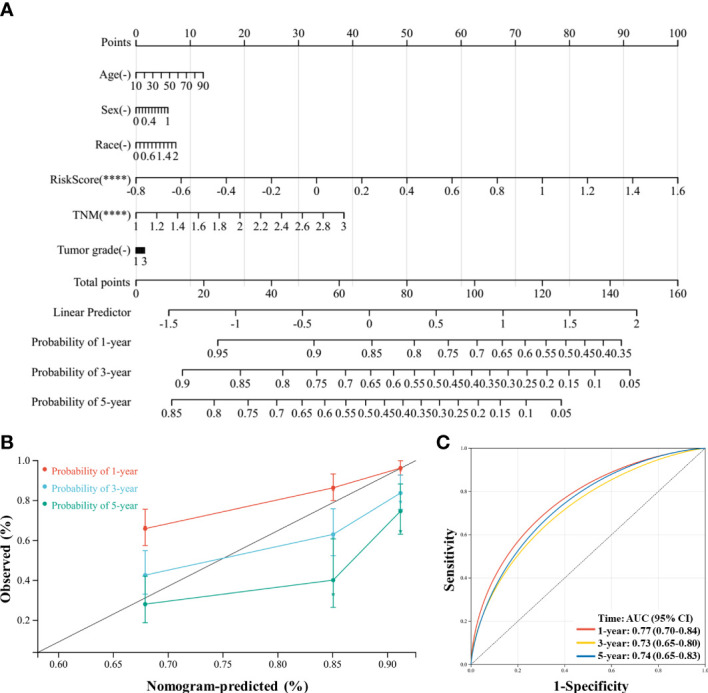
A nomogram was constructed to predict the probability of the 1-year, 3-year, and 5-year overall survival (OS) in patients with hepatocellular carcinoma (HCC). **(A)** The nomogram was constructed based on six factors, and the results suggest that the risk score and TNM (tumor, nodes, metastases) stage significantly affected the OS of patients with HCC. For the factor sex, 0 represents male and 1 represents female; for the factor race, 0 represents white, 1 represents Asian, and 2 represents others; for TNM, 1 represents stage I, 2 represents stage II, and 3 represents stages III and IV. **(B)** Calibration plots of the nomogram for the 1-year, 3-year, and 5-year OS. **(C)**, Receiver operating characteristic (ROC) curve of the nomogram prediction model. ****P < 0.0001.

Finally, a novel prognostic nomogram to predict the survival of patients with HCC was constructed by integrating risk score, age, sex, race, TNM stage, and tumor grade. Both risk score and TNM stage significantly influenced the survival of patients (both *P* < 0.05), and the risk score had a greater influence than the TNM stage (**Figure 9A**). Moreover, the calibration plots and ROC curves suggested that the model could reliably predict the OS of patients with HCC (**Figures 9B, C**).

## 4 Discussion

As a critical bioinorganic element, Cu plays important roles in various biological processes in vertebrates (15, 37), and Cu homeostasis is tightly regulated within the body. However, elevated serum and tumor levels of Cu are common in many cancers, and studies have shown that Cu plays critical roles in tumor growth and immune resistance (18, 34, 38, 39). Recently, the mechanism of Cu in regulating HCC development has become a topic of interest (40–42). For instance, a newly published study showed that elevated intracellular levels of Cu promoted the radioresistance of HCC cells, and novel treatment strategies can recover the sensitivity to radiotherapy by disrupting Cu–Fe homeostasis in HCC cells (42). Davis et al. found that the expression of Cu transporter genes was significantly altered in HCC; by limiting Cu homeostasis, the growth of HCC cell lines could be inhibited (40). Therefore, exploring the relationships among Cu metabolism, cuproptosis, and tumor immune response may provide novel insights on cancer therapy.

In our study, we systematically analyzed 59 genes involved in Cu metabolism and cuproptosis in patients with HCC from two public datasets. Results suggested that 15 CRGs significantly influenced the prognosis of patients. Furthermore, we successfully constructed a Lasso model and nomogram to predict the risk of death for patients with HCC based on the screened CRGs and 11 critical genes that were identified using the Lasso model. HCC samples were validated and potential targets that are closely associated with ICGs and immune cells, such as *PRNP*, *SNCA*, and *COX17*, were identified. Collectively, these findings confirm the key roles of CRGs in mediating tumor development, and this prediction model could help clinicians predict the prognosis of patients with HCC more easily.

The role of Cu in regulating tumor immune function and immune checkpoints has rarely been explored. In 1981, a study reported that mice fed a Cu-deficient diet made significantly fewer antibody-producing cells and had an impaired immune system (43). Another study revealed that endogenous Cu was involved in the mediation of inflammatory responses (44). In 2020, Voli et al. reported that intratumoral Cu modulated PD-L1 expression, tumor immune cell infiltration, and immune escape in neuroblastoma. However, to the best of our knowledge, studies regarding Cu metabolism and immune function in HCC are lacking. Considering this, we focused our analyses on understanding the mechanisms underlying the effects of CRGs on immune-related pathways. By performing multiple function enrichment analyses, we identified that the immune-related pathways were significantly enriched, such as complement activation-related pathways, humoral immune response, and immune response-regulating signaling pathways. Furthermore, tumor immune cell infiltration analysis showed that *ATP7A*, *ATP13A2*, and *SNCA*, which were unfavorable for the OS of patients with HCC, were positively correlated with multiple types of immune cell infiltration, whereas *COX17*, *F5*, and *ALB*, which were favorable for the OS of patients with HCC, were negatively correlated with immune cell infiltration in HCC. These results could be explained by the complexity and heterogeneity of immune contexture in HCC. Higher levels of immune cell infiltration may be associated with worse prognosis of patients with HCC owing to the accumulation of numerous suppressive immune cells, such as TAMs, exhausted T cells, and MDSCs. For instance, Wu et al. validated a simple myeloid signature known as MRS for HCC and discriminated HCC immune subtypes as immunocompetent, immunodeficient, and immunosuppressive subtypes (32). They found that the infiltration level of CD8^+^ T cells was comparable in the immunocompetent and immunosuppressive subtypes, while most T cells were PD-1^high^ exhausted T cells in the immunosuppressive subtypes, suggesting the presence of a highly immunosuppressive tumor microenvironment in patients with HCC with a high MRS.

Immune checkpoints play critical roles in regulating immune cell function and tumor immune cell infiltration, and ICI therapy has revolutionized the treatment of advanced malignancies and other diseases in recent years (45, 46). For example, Wang et al. found that increased PD-L1 expression in human neutrophils delays cellular apoptosis by triggering PI3K-dependent AKT phosphorylation, thereby promoting lung injury and increasing mortality during clinical and experimental sepsis (45). Additionally, ICIs targeting PD-1, PD-L1, or CTLA4 have enabled the possibility of long-term survival in patients with tumors such as melanoma, HCC, breast cancer, and colorectal cancer (47, 48). Previous studies demonstrated that tumor cell-intrinsic ICGs regulated tumor development (34, 49, 50). Therefore, correlations between CRGs and ICGs at the mRNA level were investigated and discussed in our study. Among the 15 critical prognostic genes, *ATP7A*, *ATP13A2*, *SNCA*, and *PRNP* were significantly positively correlated with the expression of ICGs, whereas *COX17* and *F5* were negatively correlated with the expression of ICGs. These results were consistent with those of the survival analysis in the two datasets, suggesting that CRGs influence tumor immune escape by regulating the expression of ICGs. Furthermore, based on the co-expression analysis between CRGs and ICGs, we hypothesized that infiltrated immune cells may be disabled by the high levels of immune checkpoints in tumor cells.

The meaningful findings of our study are as follows: 1) We found potential associations between CRGs and immune function regulation in HCC. Furthermore, we found that CRGs were correlated with the expression of PD-1, PD-L1, and CTLA4, which implies possible effects on regulating the immune escape and TIM, and may be promising targets to improve the efficacy of immunotherapy in HCC. To target these potential genes may cooperate with ICIs to suppress tumor growth. 2) We identified critical CRGs that significantly influence the survival of patients with HCC. We constructed a useful tool to predict the risk of death for patients with HCC based on the prognostic genes identified. 3) We analyzed the effect of cuproptosis on HCC and found that some CRGs, such as *ATP7A* and *SLC31A1*, significantly affected the OS of patients with HCC, suggesting that cuproptosis is involved in HCC progression. Moreover, cuproptosis may provide new research directions and targets for HCC clinical treatments, similar to ferroptosis. Finally, our study revealed complex functions of Cu in regulating the TIM, immune cell infiltration, and ICG expression.

Limitations of the present study are worth noting. First, *in vivo* or *in vitro* experiments are required to validate the enrichment of immune-related signaling pathways observed in the GSEA and KEGG pathway enrichment analysis. Nevertheless, some validations were performed on HCC samples in our study, and this provides a meaningful direction for scientists to further investigate the relationship between Cu metabolism and tumor immune response in HCC. Second, it was unclear whether immune escape and immune therapy resistance could be reversed by targeting the critical CRGs, although correlations were identified at the mRNA level among CRGs, tumor immune cell infiltration, and immune checkpoints. Third, multicenter clinical trials with large sample sizes are required to validate and improve our prognostic model.

## 5 Conclusion

Our study provides meaningful insight into the key roles of CRGs in the development of HCC. Functional enrichment and pathway analysis suggest a close relationship between CRGs and immune-related pathways in HCC. Critical CRGs, particularly *PRNP*, *SNCA*, and *COX17*, may influence the infiltration of multiple immune cells in HCC, and significant correlations with the expression of *PD-1*, *PD-L1*, and *CTLA4* were also observed. Collectively, CRGs could be promising therapeutic targets for HCC by regulating the TIM and immune checkpoints.

## Data availability statement

The original contributions presented in the study are included in the article/**Supplementary Material**. Further inquiries can be directed to the corresponding authors.

## Ethics statement

This study was approved by the Renji Hospital Ethics Committee (KY2020-185). The patients/participants provided their written informed consent to participate in this study.

## Author contributions

conceptualization, JT, WY, and HS; methodology, XW and JT; software, XW, DC, and YS; validation, JL and YZ; formal analysis, XW and XY; investigation, JT; resources, XW, C.Z., and JT; data curation, XW and HS; writing—original draft preparation, XW and JT; writing—review and editing, WY and JT; supervision, WY and JT; funding acquisition, DC, XY, WY, and JT. All authors contributed to the article and approved the submitted version.

## Supplementary material

Supplementary Figure 1 Study design flow chart and validation process.

Supplementary Figure 2 Comparison of the expression levels of complement activation-related genes between the two groups in the ICGC-LIRI-JP dataset.

Supplementary Figure 3 Differential pathway analysis in the TCGA-LIHC dataset grouped by risk score. **(A)** Top 20 KEGG differential pathways between two groups. **(B)** Top 20 GO-BP differential pathways between two groups.

Supplementary Figure 4 Correlation analysis between cuproptosis-related genes (CRGs) and immune checkpoint genes (ICGs). **(A)** Correlation analysis between ATP7A and ICGs. **(B)** Correlation analysis between ATP13A2 and ICGs. **(C)** Correlation analysis between F5 and ICGs.

Supplementary Figure 5 Correlation analysis between cuproptosis-related genes (CRGs) and immune cell markers. **(A)** Correlation analysis between PRNP and immune cell markers. **(B)** Correlation analysis between SNCA and immune cell markers. **(C)** Correlation analysis between COX17 and immune cell markers.
