# Supplementary figures and images for: Copper and cuproptosis-related genes in hepatocellular carcinoma: therapeutic biomarkers targeting tumor immune microenvironment and immune checkpoints
Source: Front Immunol. 2023 Apr 20;14:1123231. doi: 10.3389/fimmu.2023.1123231 (PMC10157396; doi:10.3389/fimmu.2023.1123231)

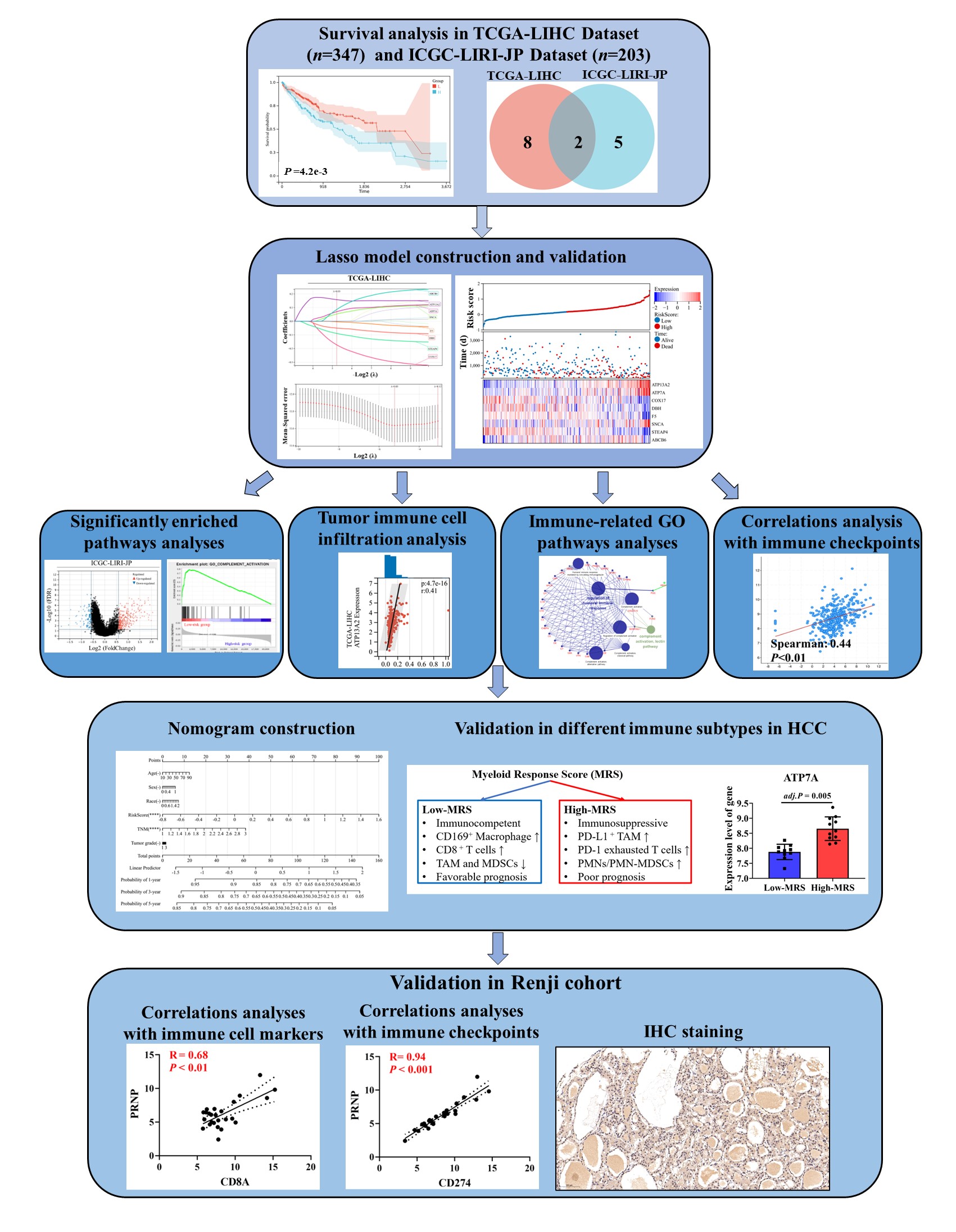

Supplement: Supplementary Figure 1 — Study design flow chart and validation process. [file Image_1.jpeg]

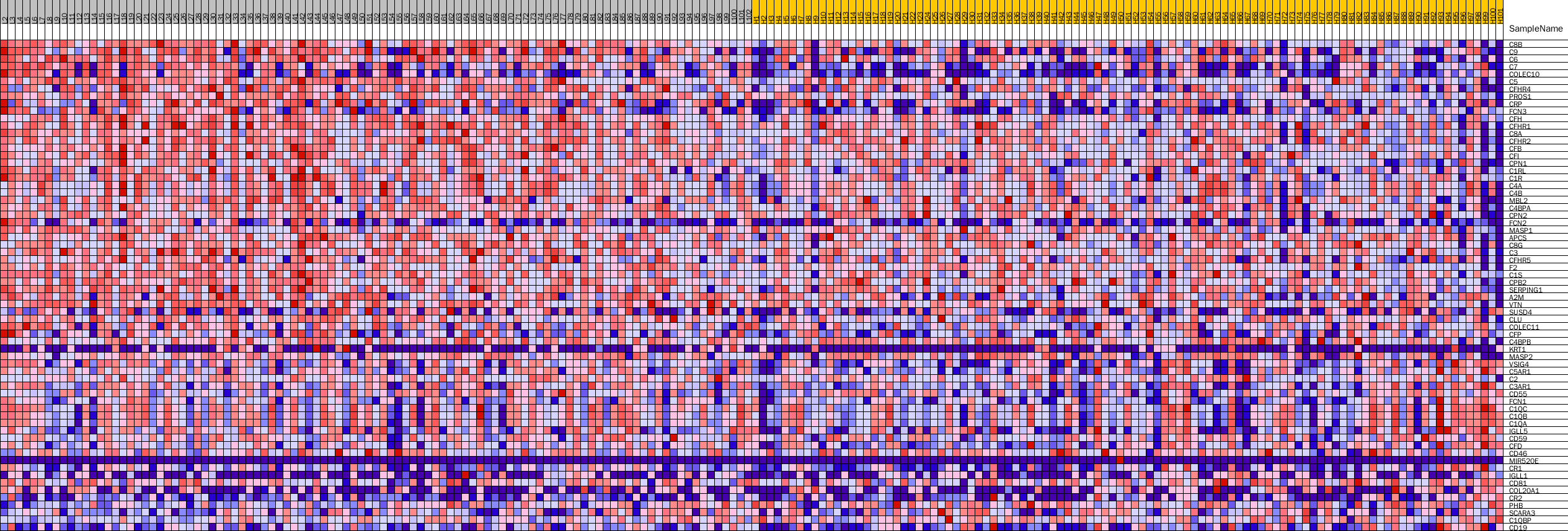

Supplement: Supplementary Figure 2 — Comparison of the expression levels of complement activation-related genes between the two groups in the ICGC-LIRI-JP dataset. [file Image_2.png]

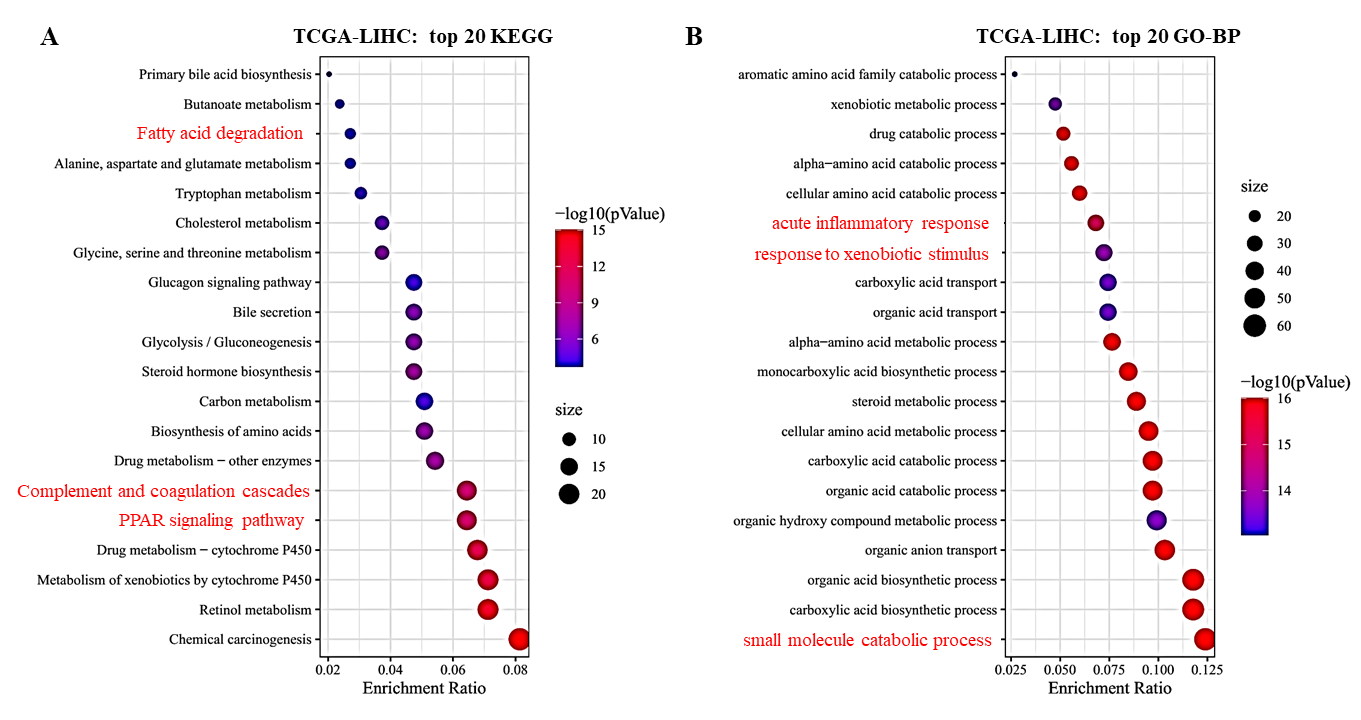

Supplement: Supplementary Figure 3 — Differential pathway analysis in the TCGA-LIHC dataset grouped by risk score. (A) Top 20 KEGG differential pathways between two groups. (B) Top 20 GO-BP differential pathways between two groups. [file Image_3.tif]

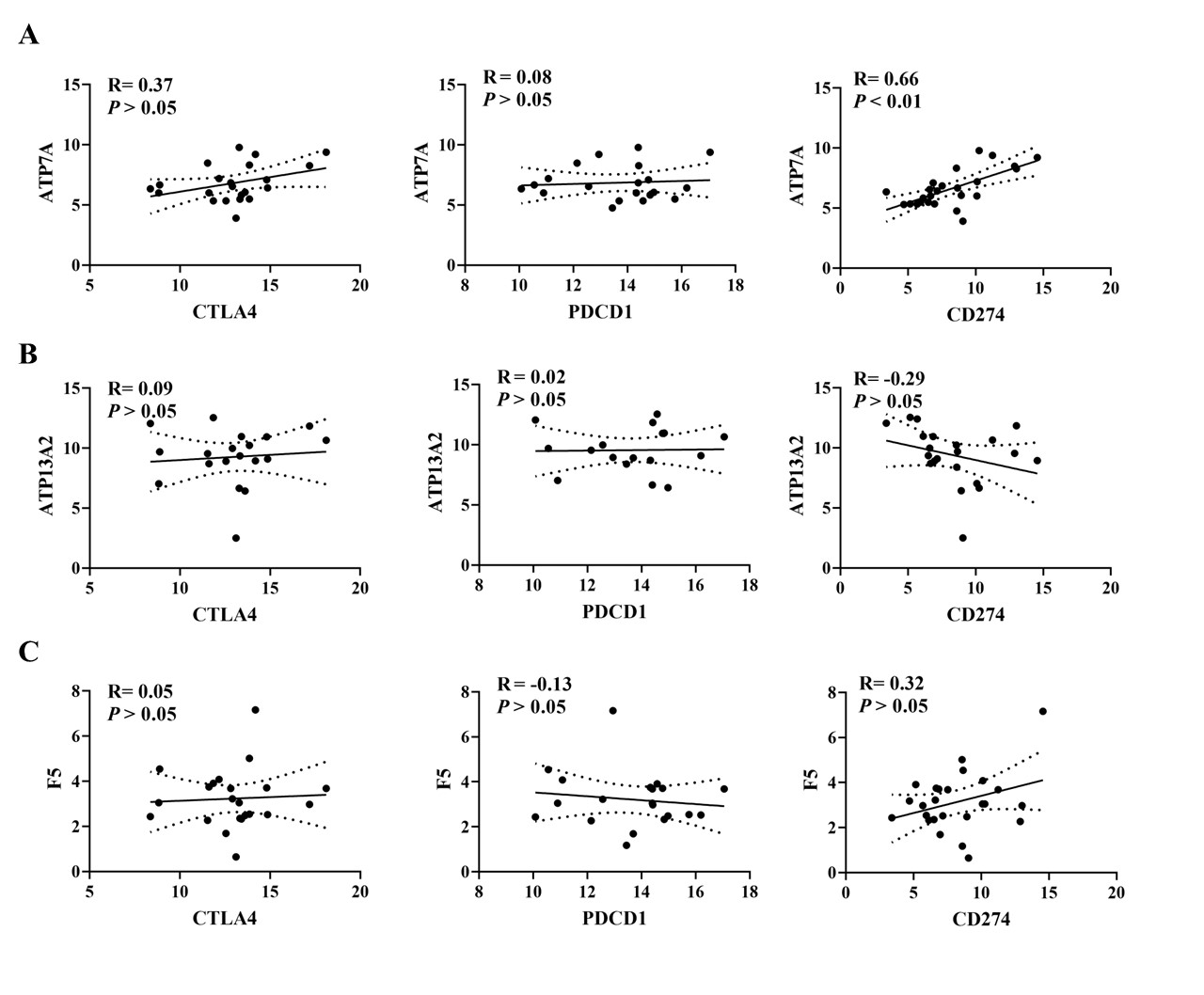

Supplement: Supplementary Figure 4 — Correlation analysis between cuproptosis-related genes (CRGs) and immune checkpoint genes (ICGs). (A) Correlation analysis between ATP7A and ICGs. (B) Correlation analysis between ATP13A2 and ICGs. (C) Correlation analysis between F5 and ICGs. [file Image_4.jpeg]

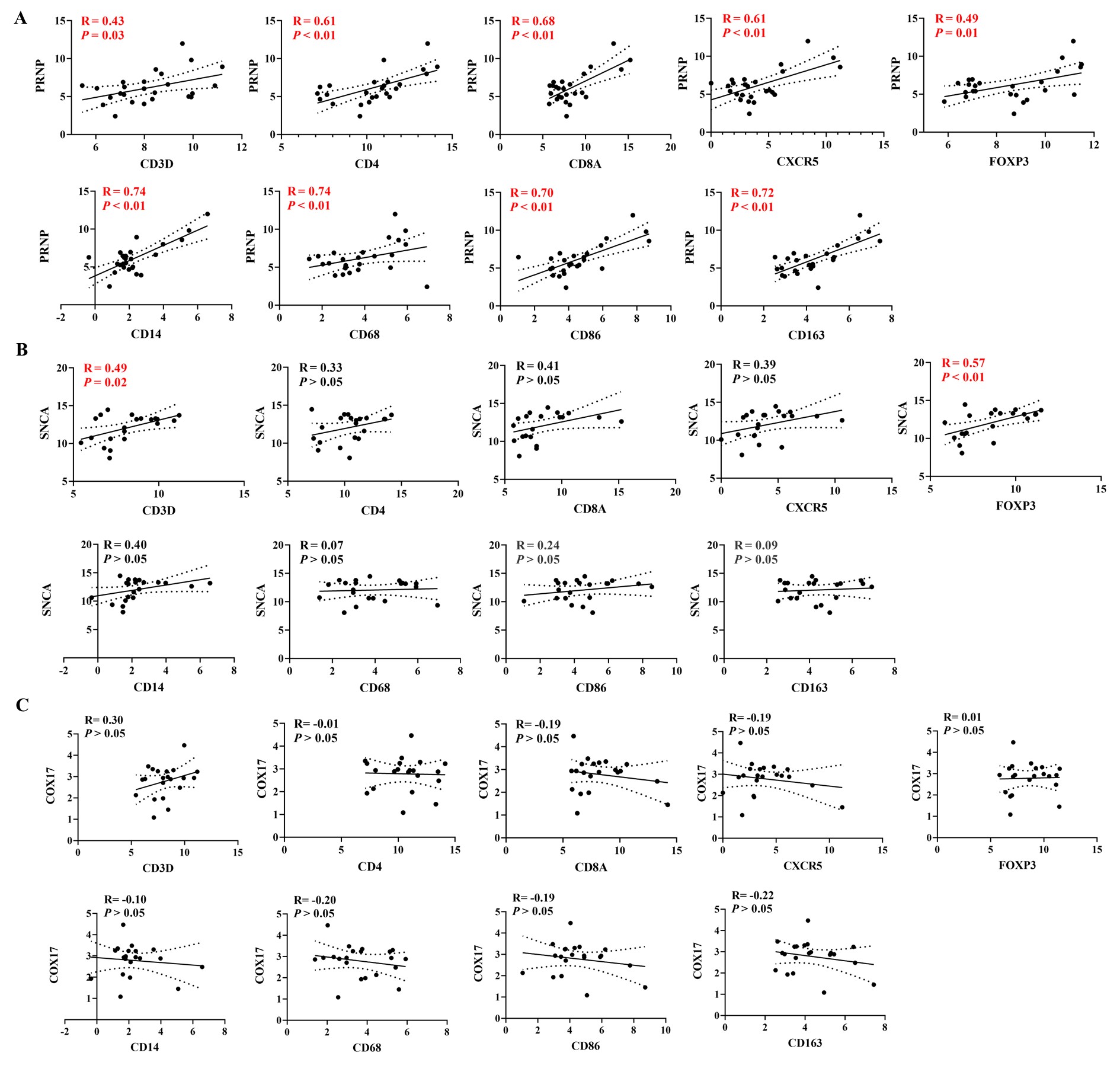

Supplement: Supplementary Figure 5 — Correlation analysis between cuproptosis-related genes (CRGs) and immune cell markers. (A) Correlation analysis between PRNP and immune cell markers. (B) Correlation analysis between SNCA and immune cell markers. (C) Correlation analysis between COX17 and immune cell markers. [file Image_5.jpeg]
