# Supplementary material for: Copper and cuproptosis-related genes in hepatocellular carcinoma: therapeutic biomarkers targeting tumor immune microenvironment and immune checkpoints
Source: Front Immunol. 2023 Apr 20;14:1123231. doi: 10.3389/fimmu.2023.1123231 (PMC10157396; doi:10.3389/fimmu.2023.1123231)
Supplement: Supplementary file 6 [file Table_1.docx]

Supplementary Table 1. List and function of 62 Cu and Cuproptosis-related genes.

| Gene symbol | Cu-related function (Annotations from Gene ontology) |
| --- | --- |
| AANAT | response to copper ion |
| ABCB6 | cellular copper ion homeostasis |
| ACR | copper ion binding |
| ADNP | copper ion binding |
| ALB | copper ion binding |
| ANKRD9 | cellular copper ion homeostasis |
| AOC1 | cellular response to copper ion; copper ion binding; cellular response to copper ion starvation |
| AOC2 | copper ion binding |
| AOC3 | copper ion binding |
| AQP1 | cellular response to copper ion |
| ARF1 | cellular copper ion homeostasis |
| ATP13A2 | cupric ion binding |
| ATP7A | copper ion export; detoxification of copper ion; cuprous ion binding; copper ion import; cellular response to copper ion; copper ion homeostasis; cellular copper ion homeostasis; essential genes for Cuproptosis |
| ATP7B | cellular copper ion homeostasis; copper transport; essential genes for Cuproptosis |
| BECN1 | cellular response to copper ion |
| CCDC22 | cellular copper ion homeostasis |
| COA6 | copper ion binding |
| COX17 | copper ion transport; copper ion binding; cuprous ion binding; copper chaperone activity |
| COX19 | cellular copper ion homeostasis |
| CP | copper ion transport; copper ion binding |
| CUTA | copper ion binding; |
| CUTC | copper ion transport; copper ion binding; copper ion homeostasis |
| DAXX | cellular response to copper ion |
| DBH | copper ion binding |
| F5 | copper ion binding |
| F8 | copper ion binding |
| HAMP | copper ion binding |
| HEPH | copper ion transport; copper ion binding |
| LOXL1 | copper ion binding |
| LOXL4 | copper ion binding |
| MAP1LC3 | cellular response to copper ion |
| MT1B | detoxification of copper ion; cellular response to copper ion |
| MT1H | detoxification of copper ion; cellular response to copper ion |
| MT1HL1 | detoxification of copper ion; cellular response to copper ion |
| MT2A | detoxification of copper ion; cellular response to copper ion; cellular copper ion homeostasis |
| MT3 | detoxification of copper ion; cellular response to copper ion |
| MT4 | detoxification of copper ion; cellular response to copper ion |
| MT-CO1 | response to copper ion |
| MT-CO2 | copper ion binding |
| NFE2L2 | cellular response to copper ion |
| OR5AR1 | copper ion binding |
| PAM | copper ion binding |
| PRND | cellular copper ion homeostasis; copper ion binding |
| PRNP | cellular copper ion homeostasis; copper ion binding; cupric ion binding; cuprous ion binding |
| SCO1 | cellular copper ion homeostasis; copper ion binding; copper chaperone activity |
| SCO2 | cellular copper ion homeostasis; copper ion binding; copper chaperone activity |
| SLC11A2 | copper ion transmembrane transport |
| SLC31A1 | copper ion transport; cellular copper ion homeostasis; copper ion transmembrane transporter activity; copper ion import; essential genes for Cuproptosis |
| SLC31A2 | copper ion transport; cellular copper ion homeostasis; regulation of copper ion transmembrane transport; copper ion transmembrane transporter activity; essential genes for Cuproptosis |
| SNCA | copper ion binding; cuprous ion binding; cellular response to copper ion |
| SNCB | cuprous ion binding |
| SOD1 | copper ion binding; response to copper ion |
| STEAP2 | cupric reductase activity; copper ion import across plasma membrane; copper ion import |
| STEAP4 | cupric reductase activity; copper ion import |
| SUMF1 | cupric ion binding |
| TFRC | response to copper ion |
| XIAP | copper ion homeostasis |
| FDX1 | essential genes for Cuproptosis |
| LIAS | essential genes for Cuproptosis |
| DLAT | essential genes for Cuproptosis |
| DLD | essential genes for Cuproptosis |
| LIPT1 | essential genes for Cuproptosis |
