# Supplementary material for: Copper and cuproptosis-related genes in hepatocellular carcinoma: therapeutic biomarkers targeting tumor immune microenvironment and immune checkpoints
Source: Front Immunol. 2023 Apr 20;14:1123231. doi: 10.3389/fimmu.2023.1123231 (PMC10157396; doi:10.3389/fimmu.2023.1123231)
Supplement: Supplementary file 7 [file Table_2.docx]

Supplementary Table 2: The sequences of primers.

| Gene | Species | Sequences |
| --- | --- | --- |
| ACTB | Human | forward 5’- CATGTACGTTGCTATCCAGGC -3’  reverse 5’- CTCCTTAATGTCACGCACGAT -3’ |
| PDCD1 | Human | forward 5’- CCAGGATGGTTCTTAGACTCCC -3’  reverse 5’- TTTAGCACGAAGCTCTCCGAT -3’ |
| CD274 | Human | forward 5’- GCTGCACTAATTGTCTATTGGG -3’  reverse 5’- CACAGTAATTCGCTTGTAGTCG -3’ |
| CTLA4 | Human | forward 5’- CAGTTAGTTCGGGGTTGTTTTT -3’  reverse 5’- TTTTCACATTCTGGCTCTGTTG -3’ |
| PRNP | Human | forward 5’- GGAACAAGCCGAGTAAGCTAAAAACCAACATGAAGCAC -3’  reverse 5’- GGTTGTGGTGACCGCGTGCTGCTTGATTG -3’ |
| SNCA | Human | forward 5’- AAGAGGGTGTTCTCTATGTAGGC -3’  reverse 5’- GCTCCTCCAACATTTGTCACTT -3’ |
| COX17 | Human | forward 5’- AGGAGAAGAAGCCGCTGAAG -3’  reverse 5’- GGCCTCAATTAGATGTCCACAGT -3’ |
| ATP7A | Human | forward 5’- GCTACCTTGTCAGACACGAATGAG -3’ |
|  |  | reverse 5’- TCTTGAACTGGTGTCATCCCTTT -3’ |
| ATP13A2 | Human | forward 5’- CACCTCGAGCATGGCCAGTATTGA -3’  reverse 5’- TGGGCCAGGGTCAGGAAGTAGC -3’ |
| F5 | Human | forward 5’- GACGTTTGACAAGCAAATCGTG -3’ |
|  |  | reverse 5’- CATTAGGGATGATGACTGGCTC -3’ |
