# Supplementary material for: Copper and cuproptosis-related genes in hepatocellular carcinoma: therapeutic biomarkers targeting tumor immune microenvironment and immune checkpoints
Source: Front Immunol. 2023 Apr 20;14:1123231. doi: 10.3389/fimmu.2023.1123231 (PMC10157396; doi:10.3389/fimmu.2023.1123231)
Supplement: Supplementary file 8 [file Table_3.docx]

Supplementary Table 3. ESTIMATE analysis of crucial CRGs in TCGA-LIHC.

| Gene | Stromal score | | Immune score | | ESTIMATE score | |
| --- | --- | --- | --- | --- | --- | --- |
|  | *P* value | R | *P* value | R | *P* value | R |
| ATP7A | 0.01 | 0.13 | < 0.001 | -0.32 | 0.17 | 0.07 |
| ATP13A2 | <0.001 | 0.19 | <0.001 | 0.31 | < 0.001 | 0.29 |
| PRNP | <0.001 | 0.3 | <0.001 | 0.3 | <0.001 | 0.32 |
| SNCA | <0.001 | 0.47 | <0.001 | 0.23 | <0.001 | 0.36 |
| COX17 | <0.001 | -0.24 | 0.002 | -0.16 | <0.001 | -0.21 |
| F5 | <0.001 | -0.25 | <0.001 | -0.23 | <0.001 | -0.26 |
| ALB | 0.34 | -0.05 | 0.39 | -0.04 | 0.33 | -0.05 |
